# Maximum likelihood multi-user MIMO detection with blind modulation classification

**DOI:** 10.1038/s41598-026-51554-3

**Published:** 2026-05-11

**Authors:** Peng Wang, Eryi Hu

**Affiliations:** Information Institute, Ministry of Emergency Management of the PRC, Beijing, 100029 China

**Keywords:** Engineering, Mathematics and computing

## Abstract

Multi-User MIMO (MU-MIMO) detection plays a pivotal role in modern wireless receivers, yet practical downlink deployments are severely bottlenecked when co-scheduled users employ unknown and highly heterogeneous modulation formats. This paper introduces a joint architecture that seamlessly integrates blind modulation classification with an adaptive non-linear MIMO detector. First, to overcome the latency of exhaustive classification, we propose a DMRS-anchored selective inference mechanism that mathematically guarantees high-fidelity priors while achieving an $85\%$ reduction in computational overhead. Subsequently, we formulate an adaptive lattice transformation that actively absorbs the geometric asymmetry of the diverse multi-user signals. By mapping these non-uniform constellations into a standardized integer search space, this mechanism enables an improved sphere decoding (SD) framework. We theoretically prove that this architecture reduces the node-expansion complexity to strictly $\mathscr {O}(1)$ per layer, completely circumventing the layer-specific sorting bottlenecks of conventional SD methods. Finally, 3GPP-compliant link-level simulations confirm that the proposed soft-output detector tightly bounds the ideal exact-ML performance in terms of both un-coded bit error rate (BER) and normalized throughput, underscoring its exceptional efficiency and reliability for practical MU-MIMO systems.

## Introduction

Multi-User MIMO (MU-MIMO) systems, which allow resource sharing among multiple users, enable full multiplexing of spatial domain resources^1^. In downlink (DL) MU-MIMO transmission, the base station (BS) can transmit signals to multiple user equipments (UEs) simultaneously. Specifically, the DL data of multiple UEs are multiplexed on the same physical resource blocks (PRBs), thereby effectively improving system capacity. Unfortunately, inter-user interference (IUI) is an inherent concomitant problem^2^. The mitigation of IUI directly affects system performance, which in turn motivates in-depth research on MU-MIMO-based receivers.

Multi-User MIMO (MU-MIMO) has been incorporated into the New Radio (NR) specifications of the 3rd Generation Partnership Project (3GPP). Its implementation involves applying a precoding matrix, specifically one derived from downlink (DL) channel state information (CSI) fed back by each user equipment (UE), to alleviate inter-user interference (IUI)^3^. However, the precoding matrix suffers from imperfections stemming from quantization errors in CSI feedback, which in turn leave residual interference in the desired signal; more precisely, signals intended for other UEs cannot be fully suppressed. Therefore, the prevalent zero-forcing and minimum mean square error (MMSE) algorithms, while featuring low computational complexity, exhibit only mediocre performance^4^. To address this, MMSE-interference rejection combining (MMSE-IRC) was developed by refining the regularization term of the MMSE framework based on interference signal characteristics^5^. Further, Enhanced-MMSE-IRC (E-MMSE-IRC) achieves additional performance gains when channel information of co-scheduled UEs is available^6^. Representative suboptimal nonlinear detectors include generalized sphere decoding (SD), which depends on generalized Cholesky factorization, and successive interference cancellation (SIC)–a technique that integrates a feedback loop into linear detection^7,8^. In general, nonlinear detectors outperform their linear equivalents, though this performance edge is accompanied by higher computational complexity.

In practical communication systems, the non-identical modulation schemes adopted by users within a cell pose significant challenges for the design of detection algorithms. This is because the modulation schemes of co-scheduled users are unknown to the target UE unless explicitly communicated via signaling. Thus, blind modulation classification becomes indispensable prior to demodulation. Modulation classification approaches can be categorized into three main types: statistical property-based methods, probabilistic methods, and machine learning-based methods. The core idea of statistical property-based methods lies in the distinguishability of specific statistical features across different modulation schemes; typical statistical features include mean, skewness, kurtosis, variance, and fourth-order cumulants^9^. Probabilistic methods assume a statistical distribution of the received signal for a given modulation scheme, with representative approaches including maximum likelihood (ML), maximum a posteriori, and variational inference^10,11^. For machine learning-based methods, features relevant to modulation recognition are extracted using training data corresponding to various modulation schemes, thereby achieving classification^12,13^.

While existing works have extensively explored isolated modulation classification and generalized SD, simply concatenating these two components is fundamentally insufficient for practical MU-MIMO environments. Conventional SD frameworks implicitly assume uniform modulation geometries across all spatial layers. Consequently, directly feeding the diverse, non-identical modulation formats extracted from a blind classifier into a standard SD induces severe algorithmic branching and computational bottlenecks. To address this literature gap, we propose an integrated near-ML detection architecture that tightly couples physical-layer characteristics with an adaptive non-linear detector, specifically tailored for MU-MIMO systems with unknown and heterogeneous modulations.

Our work fills this gap by introducing a joint architecture that bridges blind classification with an adaptive detector. We explicitly tackle the asymmetric lattice challenge. The key contributions are as follows: (i) Channel-Coherence-Driven Selective Inference: Rather than passively employing generic ML classification, we formulate the blind modulation classification as a constrained optimization problem governed by the 2D Wiener-Khinchin channel correlation theorem. By mathematically anchoring the log-likelihood inference strictly to high-confidence resource elements within the coherence interval, we drastically decimate the search space. This bespoke physical-layer integration preserves classification accuracy while achieving an $85\%$ reduction in classification overhead. (ii) To bridge the non-uniform classification outputs with the detection stage, we develop a novel signal preprocessing framework. By seamlessly integrating a layer-specific dynamic scaling matrix ($\boldsymbol{\alpha }$) into the augmented QR decomposition, we actively absorb the geometric asymmetry of multi-user signals. This mechanism uniquely transforms heterogeneous complex constellations (e.g., QPSK and 64QAM co-existing) into a normalized, unified real-valued tree structure. (iii) Order-Independent Zigzag Enumeration: Building upon the normalized lattice, we propose an optimized node enumeration strategy for the non-linear tree search. Unlike standard K-best or Schnorr-Euchner Sphere Decoding (SE-SD) that suffer from order-dependent sorting latency, our algorithm calculates the optimal path using continuous-domain bounding. This strictly maintains a near-constant $\mathscr {O}(1)$ computational overhead for node expansion regardless of the constellation size, making the architecture highly scalable for high-order modulations. Finally, we demonstrate through 3GPP-compliant simulations that this synergy effectively eliminates the performance penalty typically associated with blind detection, achieving near-ideal throughput.

## System model

We consider a DL MU-MIMO system with one BS and *K* UEs. The BS and the *k*-th UE are equipped with *M* and *N* antennas, respectively. Without loss of generality, we focus on the received signal of the *k*-th UE, which is given by

1 $$\begin{aligned} \boldsymbol{y}=\boldsymbol{H}\Big (\boldsymbol{W}_k\boldsymbol{s}_k+\sum _{k'=1,k'\ne k}^{K}\boldsymbol{W}_{k'}\boldsymbol{s}_{k'}\Big )+\boldsymbol{n} \end{aligned}$$

where $\boldsymbol{H}\in \mathbb {C}^{N\times M}$ denotes the downlink channel matrix from the BS to the *k*-th UE, and $\boldsymbol{W}_k\in \mathbb {C}^{M\times M_k}$ is the precoding matrix for the *k*-th UE, where $\sum _{k=1}^{K}M_k=M$. $\boldsymbol{s}_k\in \mathbb {C}^{M_k}$ is the transmitted symbol vector for the *k*-th UE, which is mapped from a coded bit-stream through the complex constellation $\mathscr {O}$ (symmetric $2^{Q_k}$-QAM schemes with $Q_k$ bits per symbol and $|\mathscr {O}| = 2^{Q_k}$). $\boldsymbol{n}\in \mathbb {C}^{N}$ is the additive Gaussian noise with zero mean and covariance matrix $\boldsymbol{\Lambda }$. The desired signal of the *k*-th UE is aliased with signals for other UEs. Note that different UEs may have different transmission layers and modulation orders, i.e., dimensions of precoding matrices $\boldsymbol{W}_k$ and QAM constellations may be non-identical. This brings difficulties for detection, which we will discuss in detail later. $\boldsymbol{H}\boldsymbol{W}_k$ can be obtained by the channel estimation based on the demodulation reference signal. The signal model can be rewritten as the equivalent format

2 $$\begin{aligned} \begin{aligned} \boldsymbol{y}=\boldsymbol{H}'\boldsymbol{s}+\boldsymbol{n} \end{aligned} \end{aligned}$$

where $\boldsymbol{H}'=[\boldsymbol{H}\boldsymbol{W}_1,\boldsymbol{H}\boldsymbol{W}_2,...,\boldsymbol{H}\boldsymbol{W}_K] \in \mathbb {C}^{N\times M}$ is the augmented channel matrix. The vector $\boldsymbol{s}=[\boldsymbol{s}_1;\boldsymbol{s}_2;...;\boldsymbol{s}_K] \in \mathbb {C}^{M}$ is the combination of symbol vectors of all UEs. The corresponding bits are denoted by $x_{i,b}$, where the indices *i* and *b* refer to the *b*-th bit in the binary label of the *i*-th entry of $\boldsymbol{s}$. The bit log-likelihood ratios (LLR) of soft-input soft-output MIMO detector can be naturally obtained via the definition

3 $$\begin{aligned} \begin{aligned} L_{m,b}=\text {log}\Big (\frac{P[x_{m,b}]=1|\boldsymbol{y},\boldsymbol{H}'}{P[x_{m,b}]=0|\boldsymbol{y},\boldsymbol{H}'}\Big ) \end{aligned} \end{aligned}$$

for all *MQ* bits. The following equivalent form can be derived from Bayes’s theorem^14^

4 $$\begin{aligned} \begin{aligned} L_{m,b}=\text {log}\Big (\frac{\sum _{\boldsymbol{s}\in \mathscr {X}_{m,b}^{1}}\text {p}(\boldsymbol{y}|\boldsymbol{s},\boldsymbol{H}')\text {P}[\boldsymbol{s}]}{\sum _{\boldsymbol{s}\in \mathscr {X}_{m,b}^{0}}\text {p}(\boldsymbol{y}|\boldsymbol{s},\boldsymbol{H}')\text {P}[\boldsymbol{s}]}\Big ) \end{aligned} \end{aligned}$$

where $\mathscr {X}_{m,b}^{1}$ and $\mathscr {X}_{m,b}^{0}$ are the sets of symbol vectors that have the bit corresponding to the indices *m* and *b* equal to 1 and 0, respectively. The prior $\text {P}[\boldsymbol{s}]$ represents the prior knowledge of transmitted symbols the form of bit LLRs. Assume that the entries of noise vector $\boldsymbol{n}$ are independently and identically distributed (i.i.d.) with zero mean and variance $\sigma ^2$, then the probability density function of $(\boldsymbol{y}|\boldsymbol{s},\boldsymbol{H}')$ can be simplified as

5 $$\begin{aligned} \begin{aligned} \text {p}(\boldsymbol{y}|\boldsymbol{s},\boldsymbol{H}')=\frac{1}{(\pi \sigma ^2)^N}\text {exp}\Big (-\frac{\Vert \boldsymbol{y}-\boldsymbol{H}'\boldsymbol{s}\Vert ^2}{\sigma ^2}\Big ). \end{aligned} \end{aligned}$$

With the Max-log approximation, the intrinsic LLRs can be simplified as follows^15^

6 $$\begin{aligned} \begin{aligned} L_{m,b}&=\min _{\boldsymbol{s}\in \mathscr {X}_{m,b}^0}\{\frac{1}{\sigma ^2}\Vert \boldsymbol{y}-\boldsymbol{H}'\boldsymbol{s}\Vert ^2-\text {log}\text {P}[\boldsymbol{s}]\}-\min _{\boldsymbol{s}\in \mathscr {X}_{m,b}^1}\{\frac{1}{\sigma ^2}\Vert \boldsymbol{y}-\boldsymbol{H}'\boldsymbol{s}\Vert ^2-\text {log}\text {P}[\boldsymbol{s}]\}. \end{aligned} \end{aligned}$$

For the convenience of explanation, define the partial symbol vector $\boldsymbol{s}^i=[s_i,...,s_M]^{\text {T}}$ and distance $d(\boldsymbol{s})=\frac{1}{\sigma ^2}\Vert \hat{\boldsymbol{y}}-\boldsymbol{H}'\boldsymbol{s}\Vert ^2-\text {log}\text {P}[\boldsymbol{s}]$. Reasonably assume the independence of symbols between layers of the input prior, we have^14^

7 $$\begin{aligned} \begin{aligned} \text {log}\text {P}[\boldsymbol{s}]=\sum \nolimits _{m=1}^{M}\text {log}\text {P}[s_m|\boldsymbol{s}^{m+1}]=\sum \nolimits _{m=1}^{M}\text {log}\text {P}[s_m] \end{aligned} \end{aligned}$$

where $$\text {log}\text {P}[s_m] \approx \sum _{b=1}^{Q}\frac{1}{2}(x_{m,b}L_{m,b}^A-|L_{m,b}^A|)$$ and $$L_{m,b}^{\text {A}}=\text {log}(\text {P}[x_{m,b}=1]/\text {P}[x_{m,b}=0])$$.

If there is no prior knowledge of the information bits. It is natural to assume that $\text {P}[x_{m,b}=1]=\text {P}[x_{m,b}=0]=\frac{1}{2}$ and we obtain $\text {log}\text {P}[\boldsymbol{s}]=0$ for any symbol vector $\boldsymbol{s}$. Therefore, the LLR can be further simplified as

8 $$\begin{aligned} \begin{aligned} L_{m,b}=\min _{\boldsymbol{s}\in \mathscr {X}_{m,b}^0}\{\frac{1}{\sigma ^2}\Vert \boldsymbol{y}-\boldsymbol{H}'\boldsymbol{s}\Vert ^2\} -\min _{\boldsymbol{s}\in \mathscr {X}_{m,b}^1}\{\frac{1}{\sigma ^2}\Vert \boldsymbol{y}-\boldsymbol{H}'\boldsymbol{s}\Vert ^2\}. \end{aligned} \end{aligned}$$

Regrettably, even with these simplifications in place, the computation of $L_{m,b}$ exhibits exponential growth with respect to either the bit length of each symbol or the number of symbols in vector $\boldsymbol{s}$. Thus, in the subsequent section, we focus on developing a method that circumvents exhaustive enumeration.

## Proposed MIMO detector

### Signal preprocessing

To facilitate the efficient zigzag search strategy for sphere decoding, it is essential to map the detection problem through three distinct variable domains: the original complex domain $\mathbb {C}^M$, the decoupled real-valued domain $\mathbb {R}^{2M}$, and the unified positive integer lattice $\mathbb {Z}^+$.

Firstly, we transition from the complex domain to the real-valued domain. The received signal model in (1) is rephrased into its real-valued equivalent model:

9 $$\begin{aligned} \begin{aligned} \overbrace{\begin{bmatrix}\mathfrak {R}(\boldsymbol{y}) \\ \mathfrak {I}(\boldsymbol{y})\end{bmatrix}}^{\tilde{\boldsymbol{y}}}=\overbrace{\begin{bmatrix}\mathfrak {R}(\boldsymbol{H}') & -\mathfrak {I}(\boldsymbol{H}') \\ \mathfrak {I}(\boldsymbol{H}') & \mathfrak {R}(\boldsymbol{H}')\end{bmatrix}}^{\tilde{\boldsymbol{H}}}\overbrace{\begin{bmatrix}\mathfrak {R}(\boldsymbol{s}) \\ \mathfrak {I}(\boldsymbol{s})\end{bmatrix}}^{\tilde{\boldsymbol{s}}} + \overbrace{\begin{bmatrix}\mathfrak {R}(\boldsymbol{n}) \\ \mathfrak {I}(\boldsymbol{n})\end{bmatrix}}^{\tilde{\boldsymbol{n}}} \end{aligned} \end{aligned}$$

where $\mathfrak {R}(\cdot )$ and $\mathfrak {I}(\cdot )$ denote the real and imaginary parts of the variables, respectively.

Since the real and imaginary parts of each QAM symbol are independently mapped to one-dimensional (1D) PAM symbols, we denote $\mathscr {X}_k^{\prime }$ as the decoupled real-valued or imaginary-valued components in the constellation diagram of the *k*-th user, i.e.,

10 $$\begin{aligned} \begin{aligned} \mathscr {X}_k^{'}=\{u=\alpha _k(2q-\sqrt{2^{Q_k}}+1):q\in \mathscr {Z}_{k}\} \end{aligned} \end{aligned}$$

with $\mathscr {Z}_{k} = \{0,1,...,\sqrt{2^{Q_k}}-1\}$. At this stage, the signal resides in a heterogeneous real-valued space bounded by the respective constellation geometries.

The transmission layers of users are permitted to be non-identical. We denote $r_k$ as the number of transmission layers for the *k*-th user, then we can establish the relationship between modulation orders and symbol values for each layer that $z_m\in \mathscr {Z}_{k}$ and $z_{m+M}\in \mathscr {Z}_{k}$ ($m=1,...,M$) if

11 $$\begin{aligned} \begin{aligned} \sum \nolimits _{i=1}^{k-1}r_k<m\le \sum \nolimits _{i=1}^{k}r_k. \end{aligned} \end{aligned}$$

Secondly, we transition from the bounded real-valued domain to the unified positive integer lattice. At this juncture, we introduce the core integration mechanism: the layer-specific dynamic scaling diagonal matrix $\boldsymbol{\alpha }$ . This matrix actively translates and scales the heterogeneous modulation formats ($Q_k$) identified by the prior blind classification stage. By applying this transformation, the disparate real-valued constellations are unified into a standard positive integer space ($z_m \in \mathbb {Z}^+$), completely eliminating layer-specific geometric boundaries. After this integrated model reconstruction, the distance $\boldsymbol{d}(\tilde{s})$ in the new $\mathbb {Z}^+$ domain can be seamlessly rephrased as:

12 $$\begin{aligned} \begin{aligned} d(\boldsymbol{\tilde{s}})&=\frac{1}{\sigma ^2}\Vert \boldsymbol{\tilde{y}}-\boldsymbol{\tilde{H}}\boldsymbol{\tilde{s}}\Vert ^2\\&=\frac{1}{\sigma ^2}\Big \Vert \boldsymbol{\tilde{y}}-\boldsymbol{\tilde{H}}\begin{bmatrix}\alpha _1(2z_1-\sqrt{2^{Q_1}}+1) \\ \vdots \\ \alpha _{K}(2z_{2M}-\sqrt{2^{Q_K}}+1)\end{bmatrix}\Big \Vert ^2\\&=\frac{1}{\sigma ^2}\Big \Vert \tilde{\boldsymbol{y}}+\boldsymbol{\tilde{H}}\boldsymbol{\alpha }\boldsymbol{v}-2\boldsymbol{\tilde{H}}\boldsymbol{\alpha }\boldsymbol{z}\Big \Vert ^2 \end{aligned} \end{aligned}$$

where $\boldsymbol{\alpha }$ is a diagonal matrix and $\boldsymbol{s}$ is a column vector satisfying13

14 $$\begin{aligned} \boldsymbol{v}=\begin{bmatrix} \bar{\boldsymbol{v}} \\ \bar{\boldsymbol{v}}\end{bmatrix}, \bar{\boldsymbol{v}}=[(\sqrt{2^{Q_1}}-1)\boldsymbol{I}_1;...,(\sqrt{2^{Q_K}}-1)\boldsymbol{I}_K] \end{aligned}$$

where $\boldsymbol{E}_k$ and $\boldsymbol{I}_k=[1,...,1]^{\text {T}}$ are the $M_k\times M_k$ dimensional identity matrix and the all-ones column vector of dimension $M_k$, respectively.

**Table 1:** Scaling factor of various modulation constellations in 3GPP and IEEE 802.ac specifications.

Modulation type	QPSK	16QAM	64QAM	256QAM	1024QAM
Modulation order	2	4	6	8	10
$\alpha$	$\frac{1}{\sqrt{2}}$	$\frac{1}{\sqrt{10}}$	$\frac{1}{\sqrt{42}}$	$\frac{1}{\sqrt{170}}$	$\frac{1}{\sqrt{682}}$

Next, we sparsify the channel matrix by performing QR decomposition on $2{\tilde{\boldsymbol{H}}}\boldsymbol{\alpha }$, which decouples the layer interference to a certain extent. It is crucial to highlight how this formulation departs from conventional SD approaches. Standard SD and K-best algorithms typically operate under the assumption of a uniform, known constellation lattice across all spatial streams. In contrast, our target scenario involves heterogeneous, dynamically classified modulations (e.g., $\boldsymbol{Q}_1=\boldsymbol{Q}_K$). The diagonal matrix $\mathbf {\alpha }$ defined in (13) serves as a lattice-normalization operator. By explicitly embedding the layer-specific dynamic scaling diagonal matrix $\boldsymbol{\alpha }$ into the augmented channel matrix before performing QR decomposition, we effectively transform a heterogeneous MU-MIMO search space–comprising various QAM orders–into a unified and normalized real-valued tree structure. This adaptive lattice transformation is the key prerequisite that allows a single recursive search to handle mixed-modulation boundaries seamlessly.

We denote $2{\tilde{\boldsymbol{H}}}\boldsymbol{\alpha }=\boldsymbol{Q}\boldsymbol{R}$, where $\boldsymbol{Q}_{2N\times 2M}$ is a unitary matrix and $\boldsymbol{R}$ is an $2M\times 2M$ upper triangular matrix with positive diagonal elements. Thus, the distance can be expressed in a more concise form as

15 $$\begin{aligned} \begin{aligned} d(\boldsymbol{z})&=\frac{1}{\sigma ^2}\Big \Vert \boldsymbol{Q}^{\text {H}}\boldsymbol{\tilde{y}}+\boldsymbol{Q}^{\text {H}}\boldsymbol{\tilde{H}}\begin{bmatrix}\alpha _1(\sqrt{2^{Q_1}}-1)\boldsymbol{I}_1 \\ \vdots \\ \alpha _K(\sqrt{2^{Q_K}}-1)\boldsymbol{I}_K\end{bmatrix}-\boldsymbol{R}\begin{bmatrix}\boldsymbol{z}_1 \\ \vdots \\ \boldsymbol{z}_K\end{bmatrix}\Big \Vert ^2\\&=\frac{1}{\sigma ^2}\Vert \hat{\boldsymbol{y}}-\boldsymbol{R}\boldsymbol{z}\Vert ^2\\ \end{aligned} \end{aligned}$$

where $\hat{\boldsymbol{y}}$ denotes the transformed signal and $\boldsymbol{R}$ denotes the channel matrix, respectively. Hence (8) assumes the normalized form

16 $$\begin{aligned} \begin{aligned} L_{m,b}=\min _{\boldsymbol{z}\in \mathscr {Z}_{m,b}^0}\{\frac{1}{\sigma ^2}\Vert \hat{\boldsymbol{y}}-\boldsymbol{R}\boldsymbol{z}\Vert ^2\} -\min _{\boldsymbol{z}\in \mathscr {Z}_{m,b}^1}\{\frac{1}{\sigma ^2}\Vert \hat{\boldsymbol{y}}-\boldsymbol{R}\boldsymbol{z}\Vert ^2\} \end{aligned} \end{aligned}$$

where $\mathscr {Z}=\prod _{k=1}^K\vert \mathscr {Z}_{Q_k}\vert ^{2M_k}$ is the set of real symbol vector $\boldsymbol{z}$. $\mathscr {Z}_{m,b}^0$ and $\mathscr {Z}_{m,b}^1$ are the sets of symbol vectors that have the *b*-th bit corresponding to the *m*-th layer symbol equal to 1 and 0, respectively. Subsequently, we put forward an enhanced sphere decoding algorithm. This method is designed to realize soft-output detection for MU-MIMO systems, while simultaneously integrating the capability of blindly estimating modulation orders among co-scheduled users.

### Detect modulation orders of co-scheduled UEs blindly

#### Blind modulation orders classification algorithm

In a MU-MIMO system, the modulation format of co-scheduled UEs remains inaccessible to the target UE in the absence of explicit signaling. As such, these formats must be reliably deduced with strong confidence prior to data detection. For each co-scheduled user, the set of potential modulation types and their corresponding constellation sizes are sourced from Table 1. We denote the modulation of all the co-scheduled user as $\boldsymbol{m}=[m_1,...,m_K]$, where $m_k$ is the modulation order of the *k*-th co-user. Therefore the constellation size of co-user *k* is $M_k=2^{m_k}$. For user *k*, the transmitted symbol vectors $\boldsymbol{s}_k$ are taken from a set of $M_k^{r_k}$ complex numbers if modulation $m_k$ is used, with $r_k$ be its transmission layer. Given a received vector $\boldsymbol{y}$, the probability that it is generated from modulation $\boldsymbol{m}$ is

17 $$\begin{aligned} \begin{aligned} \mathbb {P}(\boldsymbol{y}|\boldsymbol{m})=\sum _{j=1}^{N_{symb}}P(\boldsymbol{y}|\boldsymbol{s}^j)P(\boldsymbol{s}^j|m) \end{aligned} \end{aligned}$$

where $N_{symb}=\prod _{k=1}^{K}M_k^{r_k}$, $\boldsymbol{s}^j$ is the possible transmitted symbol vector corresponding to $\boldsymbol{y}$, $\mathbb {P}(\boldsymbol{y}|\boldsymbol{s}^j)$ denotes the probability that the transmitted vector $\boldsymbol{s}$ is actually $\boldsymbol{s}^j$ from modulation $\boldsymbol{m}$.

**Fig. 1:**
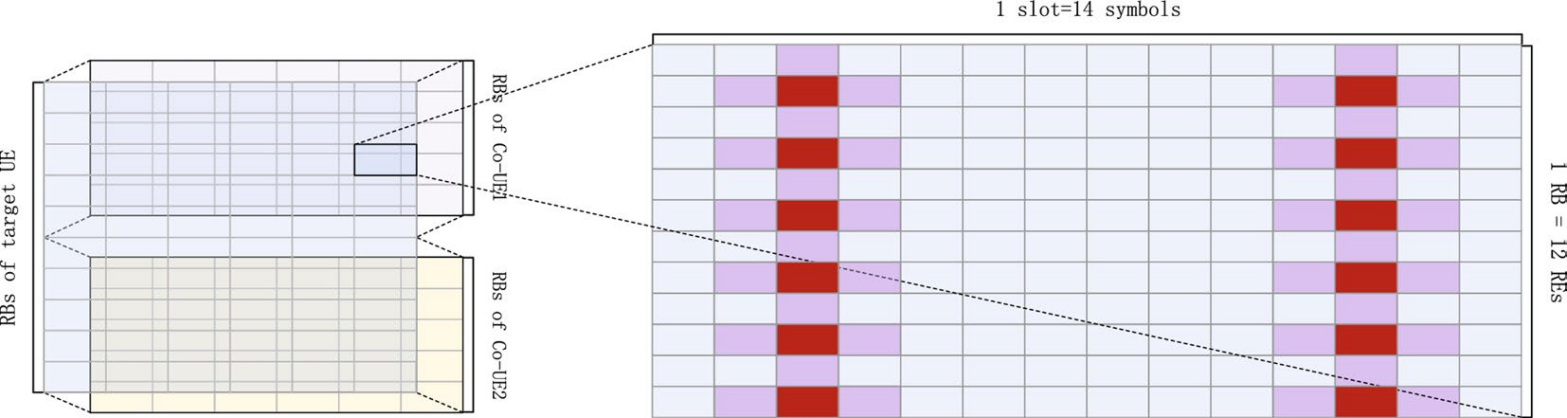
Illustration of one possible case of MU-MIMO transmission in 3GPP. There are two co-scheduled UEs, each sharing partial resource overlap with the target UE. User resource allocation uses resource blocks as the granularity, each RB including 12 REs. DMRS symbols are marked by red blocks (assuming no DMRS multiplexing). The preferred symbols for modulation classification are derived from purple blocks.

If no a priori information, these $N_{symb}$ events are equiprobable, i.e., $\mathbb {P}(\boldsymbol{s}^j|m)=\frac{1}{N_{symb}}$ for all the possible $\boldsymbol{s}^j$. Furthermore, under the premise that the signal is corrupted by white Gaussian noise, the likelihood $\mathbb {P}(\boldsymbol{y}|\boldsymbol{s}^j)$ is thereby expressed as

18 $$\begin{aligned} \begin{aligned} \mathbb {P}(\boldsymbol{y}|\boldsymbol{s}^j)=\frac{1}{\sqrt{2\pi }\sigma }\exp \Big (-\frac{\vert \boldsymbol{y}-\boldsymbol{H}^{'}\boldsymbol{s}^j\vert ^2}{2\sigma ^2}\Big ) \end{aligned} \end{aligned}$$

Therefore, the probability $P(\boldsymbol{y}|\boldsymbol{m})$ can be rewritten as

19 $$\begin{aligned} \begin{aligned} \mathbb {P}(\boldsymbol{y}|\boldsymbol{m})=\frac{1}{N_{symb}}\sum _{j=1}^{N_{symb}}\frac{1}{\sqrt{2\pi }\sigma }\exp \Big (-\frac{\vert \boldsymbol{y}-\boldsymbol{H}^{'}\boldsymbol{s}^j\vert ^2}{2\sigma ^2}\Big ) \end{aligned} \end{aligned}$$

When a plurality of samples are available, the joint probability becomes computable, and the probability of misclassification is anticipated to decline. Let the sample size be *L*, denoted as $\boldsymbol{G}=[\boldsymbol{y}_1\quad ...\quad \boldsymbol{y}_L]$. It is reasonable to assume that the *L* transmitted symbols follow an independent and identically distributed (i.i.d.) pattern; under this premise, the probability of the modulation scheme $\boldsymbol{m}$ conditional on $\boldsymbol{y}$ can be computed as

20 $$\begin{aligned} \begin{aligned} \mathbb {P}(\boldsymbol{G}|\boldsymbol{m})=\prod _{l=1}^{L}\sum _{j=1}^{N_{symb}}\frac{1}{\sqrt{2\pi }\sigma N_{symb}}\exp \Big (-\frac{\vert \boldsymbol{y}_l-\boldsymbol{H}^{'}\boldsymbol{s}^j\vert ^2}{2\sigma ^2}\Big ) \end{aligned} \end{aligned}$$

Modulation type identification can be accomplished by maximizing $\mathbb {P}(\boldsymbol{G}|\boldsymbol{m})$ across all candidate modulation formats. Put another way, the determination of the modulation scheme hinges on this maximization process

21 $$\begin{aligned} \begin{aligned} \boldsymbol{m}^{*}=\arg \max _{\boldsymbol{m}\in \mathscr {M}}\mathbb {P}(\boldsymbol{G}|\boldsymbol{m}). \end{aligned} \end{aligned}$$

where the set $\mathscr {M}=\{[m_1,...,m_K]:m_k\in \{2,4,6,8,10\},k=1,...,K\}$.

While the maximum likelihood (ML) approach delivers optimal performance, it remains computationally intractable. This is rooted in the necessity of exhaustive enumeration across all constellation points. By leveraging the monotonicity of the logarithmic function and incorporating the Max-log approximation, one can derive the equivalent log-likelihood function

22 $$\begin{aligned} \begin{aligned} \text {log}(\mathbb {P}(\boldsymbol{G}|\boldsymbol{m}))=\sum \nolimits _{l=1}^{L}\text {log}\;\mathbb {P}(\boldsymbol{y}_l|\boldsymbol{m})\approx \sum _{l=1}^{L}\Big [\max \big (-\frac{\vert \boldsymbol{y}_l-\boldsymbol{H}^{'}\boldsymbol{s^j}\vert ^2}{2\sigma ^2}\big )-\text {log}(\sqrt{2\pi }\sigma N_{symb})\Big ] \end{aligned} \end{aligned}$$

The modulation with maximum likelihood can be determined by solving the following maximization problem.

23 $$\begin{aligned} \begin{aligned} \boldsymbol{m}^{*}=\arg \max _{\boldsymbol{m}\in \mathscr {M}}\quad \text {log}(\mathbb {P}(\boldsymbol{G}|\boldsymbol{m})). \end{aligned} \end{aligned}$$

Employing this log-max approximation, the error arising from the approximation is bounded above by at most $\text {log}(N_{symb})$. Simplified near-maximum likelihood (near-ML) modulation classification, in essence, centers on the search for the minimum Euclidean distance–a process that can be carried out using the traditional SD algorithm.

Rather than uniformly processing all received symbols–which inherently introduces severe error propagation from channel aging and imposes prohibitive latency–we propose a Frame-Structure-Aware Selective Inference Mechanism. To mathematically guarantee the high-fidelity prior required by the subsequent sphere decoding, we dynamically constrain the log-likelihood maximization exclusively to a subset of high-confidence symbols. As illustrated in Fig. 1, this selective classification exploits the physical resource block (PRB) grid geometry by anchoring the inference strictly to Resource Elements (REs) adjacent to the DMRS. By tightly coupling the classification logic with the physical-layer channel coherence characteristics, this DMRS-anchored strategy mitigates the impact of channel estimation deterioration over the time slot. This bespoke physical-layer integration not only ensures a highly reliable modulation format matrix $\textbf{m}^*$ for the detection stage but also drastically truncates the computational overhead compared to conventional exhaustive classifiers.

#### Quantitative complexity and reliability analysis

Rather than uniformly processing all received symbols, we propose a Frame-Structure-Aware Selective Inference Mechanism. This approach is fundamentally grounded in modeling the degradation of the Log-Likelihood function caused by channel aging.

Let $\mathscr {G}$ denote the complete set of Resource Elements (REs) within a PRB grid, with $|\mathscr {G}| = N_{total}$. At the *n*-th RE, the received signal incorporating the channel estimation error is modeled as:

24 $$\begin{aligned} y_n = \hat{H}_n s_n + (H_n - \hat{H}_n) s_n + w_n \end{aligned}$$

where $\hat{H}_n$ is the estimated channel matrix, $s_n$ is the transmitted symbol, and $w_n \sim \mathscr{C}\mathscr{N}(0, \sigma _w^2)$ is the additive white Gaussian noise. The term $e_n = H_n - \hat{H}_n$ denotes the channel estimation error. Consequently, the effective noise variance $\sigma _{eff,n}^2$ at the *n*-th RE is amplified by this estimation inaccuracy, formulated as $\sigma _{eff,n}^2 = \sigma _w^2 + \epsilon _n E_s$, where $E_s$ is the average symbol energy, and $\epsilon _n = \mathbb {E}[||H_n - \hat{H}_n||^2]$ is the Mean Square Error (MSE) of the channel estimate^16^.

According to the Wiener-Khinchin theorem applied to WSSUS fading channels, within the coherence interval, the channel estimation MSE $\epsilon _n$ under MMSE filtering increases monotonically with the temporal offset $\Delta t_n$ and spectral offset $\Delta f_n$ from the nearest DMRS^17^. This spatial-temporal degradation is fundamentally governed by the channel’s 2D autocorrelation function:

25 $$\begin{aligned} \epsilon _n \approx \sigma _H^2 \left( 1 - |\rho (\Delta t_n, \Delta f_n)|^2 \right) \end{aligned}$$

where $\rho (\cdot )$ represents the normalized spacetime-frequency correlation function (e.g., typically modeled by the Jakes’ Doppler spectrum in the time domain).

A conventional exhaustive classifier blindly evaluates the likelihood across the entire grid $\mathscr {G}$, yielding a complexity of $\mathscr {O}(N_{total} \cdot \sum _{k=1}^K |\mathscr {M}_k|)$. However, accumulating log-likelihoods from REs with large $\epsilon _n$ introduces severe error propagation and diminishes the overall Fisher Information of the modulation classifier.

Therefore, we formulate the selective inference as a constrained optimization problem to maximize classification reliability under a strict computational budget $\mathscr {C}_{max}$:

26 $$\begin{aligned} \mathscr {G}_{sel}^* = \arg \max _{\mathscr {G}' \subset \mathscr {G}} \sum _{n \in \mathscr {G}'} \frac{1}{\sigma _{eff,n}^2} \quad \text {s.t.} \quad \mathscr {O}\left( |\mathscr {G}'| \cdot \sum _{k=1}^K |\mathscr {M}_k|\right) \le \mathscr {C}_{max} \end{aligned}$$

The mathematical solution to this objective function inherently restricts $\mathscr {G}_{sel}^*$ to the REs strictly adjacent to the DMRS, where $\epsilon _n \rightarrow 0$. By defining the decimation factor $\eta = |\mathscr {G}_{sel}^*| / |\mathscr {G}|$, our architecture scales down the operational complexity to $\mathscr {O}(\eta \cdot N_{total} \cdot \sum _{k=1}^K |\mathscr {M}_k|)$. As validated in Fig. 1, by anchoring the estimation to symbols adjacent to DMRS (yielding $\eta \approx 15\%$) the proposed detector maintains a high classification reliability while effectively delivering an $85\%$ reduction in computational overhead compared to the exhaustive baseline. This bespoke physical-layer integration ensures that the blind MC stage completely avoids becoming a latency bottleneck.

### Improved sphere decoding For MU-MIMO detection

**Fig. 2:**
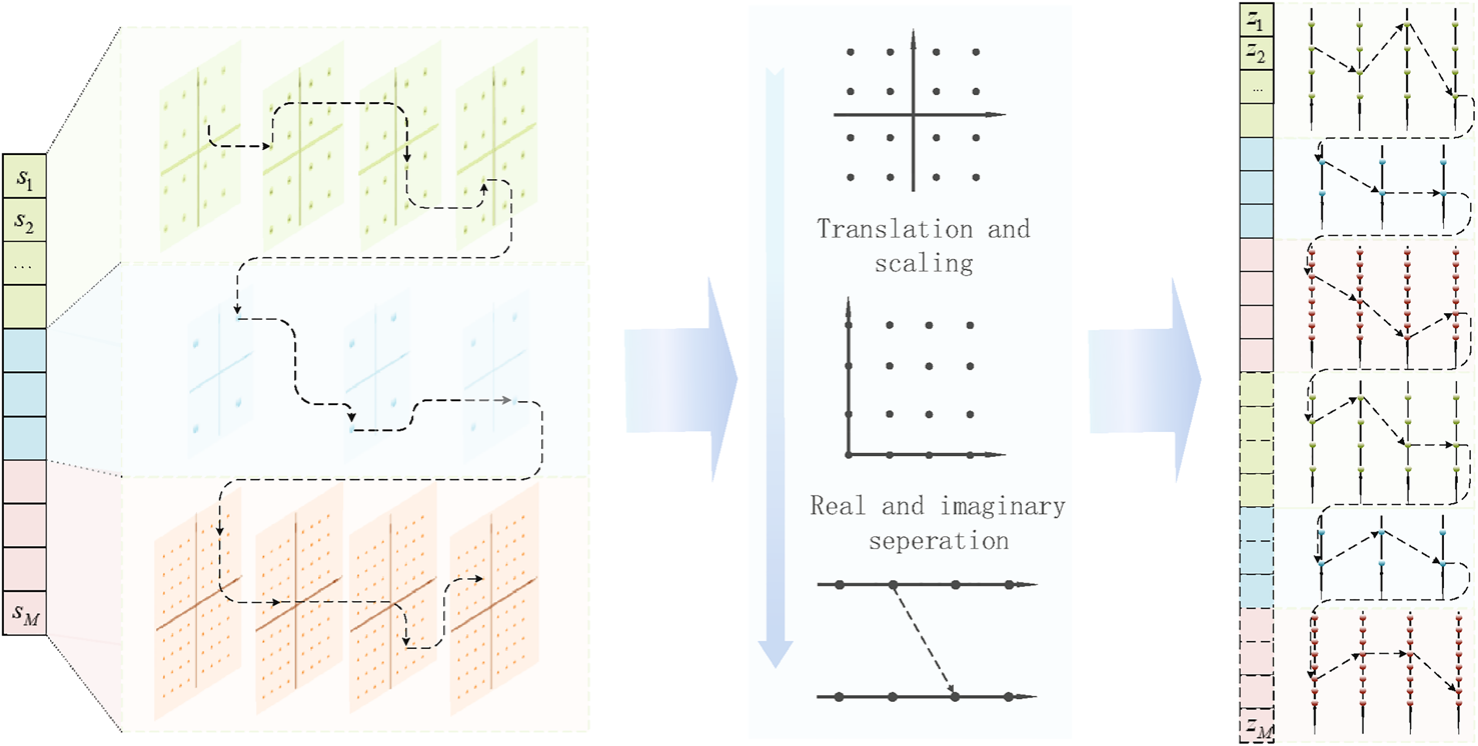
Illustration of signal preprocessing. User number $K = 3$ and the respective layers are $r_1 = 4$, $r_2 = 2$, $r_3 = 4$. The modulation orders of the 3 users are 16QAM, QPSK and 64QAM, respectively. The signal preprocessing includes symbol real-imaginary separation, constellation point shifting and scaling. So the two-dimensional QAM-seeking of 10 layers is converted to one-dimensional PAM-seeking of 20 layers, while the PAM lattices are simplified to consecutive positive integers.

We construct our detector by leveraging list sphere decoding (list-SD), with the objective of identifying a list of candidate symbol vectors that lie within a comparably small spherical radius. The bit-level log-likelihood ratios (LLRs) derived from these symbols are then employed to serve as an approximation of the ML solution. (see Fig. 2).

Exploiting the triangular form of $\boldsymbol{R}$ in (15), the $l^2$-norm of the vector $\hat{\boldsymbol{y}}-\boldsymbol{R}\boldsymbol{z}$ can be expanded as

27 $$\begin{aligned} \begin{aligned} \boldsymbol{z}_{opt} = \arg \min _{\boldsymbol{z}}\frac{1}{\sigma ^2}\sum _{m=1}^{2M}\Big \vert \hat{y}_m-\sum _{n=m}^{2M}R_{m,n}z_n\Big \vert ^2. \end{aligned} \end{aligned}$$

This process may be conceptualized as a tree-based search problem comprising 2*M* layers. SD conducts a bottom-to-top search, initiating from the 2*M*-th row. Once a symbol is identified, the next symbol in the row immediately above is detected based on this result, with this pattern continuing in sequence. Such a search procedure can be implemented recursively as

28 $$\begin{aligned} \begin{aligned} T_m(\boldsymbol{z}^m)=T_{m+1}(\boldsymbol{z}^{m+1})+\vert \Delta T_m\vert ^2\\ \end{aligned} \end{aligned}$$

29 $$\begin{aligned} \begin{aligned} \Delta T_m=\Big (\hat{y}_m-\sum \nolimits _{n=m+1}^{2M}R_{m,n}z_n\Big )-R_{m,m}z_m\\ \end{aligned} \end{aligned}$$

where $\boldsymbol{z}^m=[z_m,...,z_{2M}]^{\text {T}}$, $T_m(\boldsymbol{z}^m)$ is the accumulated partial Euclidean distance (PED) of the bottom $2M-m+1$ levels. $\vert \Delta T_m\vert ^2$ denotes the distance increment from level $m+1$ to *m* in the tree. Based on the PED updating formula, we describe the proposed algorithm as follows.

We transform the MU-MIMO detection problem into a tree with 2*M* levels. Without loss of generality, let us consider level *m* of the tree and assume that the set of *K* candidate nodes in level $m+1$ are already determined and denote by $\mathscr {K}_{m+1}$. Each node in level $m+1$ has $\sqrt{2^{Q_{k^{*}}}}$ possible children, so there are $K\sqrt{2^{Q_{k^{*}}}}$ possible children in level *m* (assume that the level *m* corresponds to user $k^*$, i.e., $\sum _{k=1}^{k^{*}-1}r_k<m\le \sum _{k=1}^{k^{*}}r_k$). Each node in level $m+1$ has $\sqrt{2^{Q_{k^*}}}$ possible children, resulting in $K\sqrt{2^{Q_{k^*}}}$ candidate children in level *m*. (Here, we assume level *m* corresponds to user $k^*$, i.e., $\sum _{i=1}^{k^*-1}r_i < m \le \sum _{i=1}^{k^*}r_i$). We refer to the nodes in level $m+1$ as parent nodes and their corresponding descendants in level *m* as children.

**Fig. 3:**
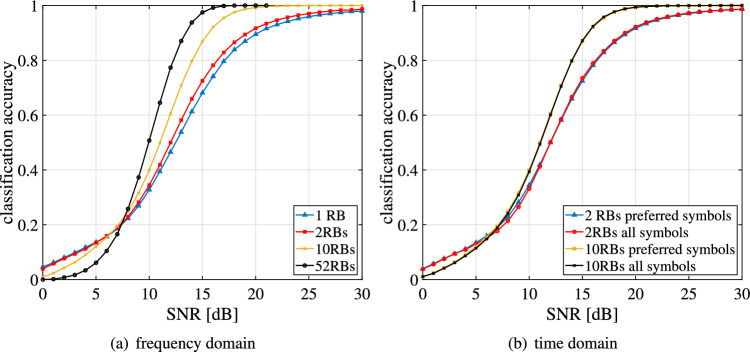
Modulation classification accuracy versus SNR. The target user receives signals interrupted by a co-scheduled user, both are configured with single layer transmission. The modulation of the co-scheduled user is set to be 16-QAM. The impact of number of RBs per inference on accuracy is depicted in (**a**), while the number of symbols within one slot per inference is changed in (**b**), where all symbols means all of the data symbols except the DMRS symbols within the slot are used for classification, preferred symbols means that only the symbols adjacent to DMRS are used.

The strategy of finding the K best children at each level efficiently is crucial. While conceptual node expansion through zigzag movements shares foundational principles with classical Schnorr-Euchner enumeration, applying standard SE directly to our scenario–where co-scheduled users possess unknown and non-uniform modulation formats–would typically require complex, layer-specific dynamic boundary checks.

However, thanks to the adaptive lattice transformation introduced in (12)-(15), all heterogeneous complex constellations are seamlessly mapped onto a unified positive integer lattice. This allows an optimized zigzag searching strategy to be used to drastically reduce the computation complexity based on (28) without being hindered by non-uniform constellation boundaries. For each node in $\mathscr {K}_{m+1}$, the first child needs to be found. Since the calculation of the first child and the next child of all nodes in $\mathscr {K}_{m+1}$ are performed independently, we focus on one node for brevity. Based on (29), the first child $z_m^{[1]}$ of a node in $\mathscr {K}_{m+1}$ is the one minimizing increment $\vert \Delta T_m\vert ^2$.

30 $$\begin{aligned} \begin{aligned} z_m^{[1]}=\arg \min _{z_m}\vert \Delta T_m\vert ^2 \end{aligned} \end{aligned}$$

to this end, we just need to round $\frac{L_m}{R_{m,m}}$ to the nearest integer value in $\mathscr {Z}_{k^{*}}$. Specifically, to determine the best children in order, one just need to take zigzag movements in $\mathscr {Z}_{k^{*}}$ starting from $z_m^{[1]}$.

31 $$\begin{aligned} \begin{aligned} z_m^{[1]}= \left\{ \begin{array}{lc} [\frac{L_m}{R_{m,m}}] & 0\le \frac{L_m}{R_{m,m}} \le \sqrt{2^{Q_{k^{*}}}} \\ \sqrt{2^{Q_{k^{*}}}} & \frac{L_m}{R_{m,m}} \ge \sqrt{2^{Q_{k^{*}}}}\\ 0 & \frac{L_m}{R_{m,m}} < 0\\ \end{array} \right. \end{aligned} \end{aligned}$$

where $[\cdot ]$ stands for the rounding operation. A notable advantage of this integrated architecture is that the computational complexity of finding the next child node is strictly $\mathscr {O}(1)$ and completely independent of the constellation order $Q_k$. Because the preceding lattice transformation normalizes the mixed modulation formats, the algorithm bypasses the exhaustive sorting overhead typically required by standard K-best or SE-SD when dealing with varying lattice sizes. This makes it a highly scalable and promising approach for next-generation MU-MIMO systems utilizing ultra-high-order modulation schemes such as 256-QAM and 1024-QAM.

Zigzag enumeration operations for all parent nodes are performed in parallel. Once each parent node produces its best child, amalgamate these *K* candidate nodes to form a pending node set $\mathscr {C}$, and the PEDs of the elements in $\mathscr {C}$ form a set $\mathscr {D}$.

32 $$\begin{aligned} \begin{aligned} \mathscr {C} =\{z_{m,1}^{[1]},...,z_{m,K}^{[1]}\} \end{aligned} \end{aligned}$$

33 $$\begin{aligned} \begin{aligned} \mathscr {D} =\{d_{m,1}^{[1]},...,d_{m,K}^{[1]}\} \end{aligned} \end{aligned}$$

where $z_{m,k}^{[l]}$ and $d_{m,k}^{[l]}$ represent the child with the *l* lowest PED of the *k*-th parent in level *m* and the corresponding PED, respectively. Sort these candidates of $\mathscr {D}$ and add the child with smallest PED into $\mathscr {K}_m$.

34 $$\begin{aligned} \begin{aligned} k'=\arg \min _k\mathscr {D} =\arg \min _k \{d_{m,1}^{[1]},...,d_{m,K}^{[1]}\} \end{aligned} \end{aligned}$$

35 $$\begin{aligned} \begin{aligned} \mathscr {K}_m = \mathscr {K}_m \cup \{z_{m,k'}^{[1]}\}. \end{aligned} \end{aligned}$$

Next, the $k'$-th parent produces its next best child $z_{m,k'}^{[2]}$ by zigzag movement, and replace $z_{m,k'}^{[1]}$ in the pending set $\mathscr {C}$ by $z_{m,k'}^{[2]}$.

36 $$\begin{aligned} \begin{aligned} \mathscr {C} = \Big (\mathscr {C}\setminus \{z_{m,k'}^{[1]}\}\Big ) \cup \{z_{m,k'}^{[2]}\} \end{aligned} \end{aligned}$$

37 $$\begin{aligned} \begin{aligned} \mathscr {D} = \Big (\mathscr {D}\setminus \{d_{m,k'}^{[1]}\}\Big ) \cup \{d_{m,k'}^{[2]}\} \end{aligned} \end{aligned}$$

then sort the elements in $\mathscr {D}$ again to find the lowest PED and add the corresponding node in $\mathscr {C}$ to $\mathscr {K}_m$. Perform these steps recursively until all *K* best nodes are filtered out, which means the search from level $m+1$ to *m* is completed.

We provide a comprehensive summary of the detector’s operational workflow in Algorithm 1. Unlike conventional SD pseudo-codes that require nested loops for boundary verification and PED array sorting, Algorithm 1 explicitly highlights the operational differences. Driven by the adaptive lattice transformation, the node expansion (Steps 10-12) replaces traditional sorting with a direct continuous-domain scalar rounding, executing strictly in $\mathscr {O}(1)$ complexity per layer. The list size $K_{list}$ serves as the pivotal algorithmic parameter. In practical deployments, the condition for setting $K_{list}$ is determined by the balance between error-correction performance and hardware-level parallelism constraints. For the comprehensive evaluations presented in this paper, $K_{list}$ is empirically initialized to a typical value of 24, which sufficiently bounds the sub-optimality gap at the target SNR. Additionally, the initial PEDs for the candidate list are initialized to infinity ($\infty$) to unconditionally accept the initial zigzag search paths.

**Algorithm 1:**
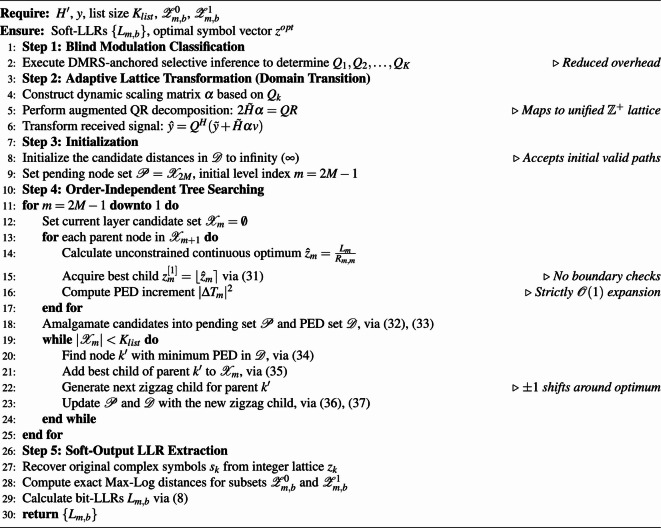
Improved Integrated SD for Soft-Output MU-MIMO Detection

#### Theoretical bounds on complexity and asymptotic optimality

To rigorously substantiate the mathematical advantages of the proposed detection architecture, we establish its computational complexity comparison and optimality bounds through the following formal propositions.

Computational Order Comparison in Heterogeneous Scenarios: To evaluate the efficiency of the proposed architecture, we first perform a computational order comparison against conventional K-best and SE-SD methods. It is worth noting that conventional enumerative SD methods implicitly assume uniform modulation geometries. If these standard methods were naively extended to the heterogeneous MU-MIMO environment targeted in this paper, the search process would require dynamic boundary checks and layer-specific step-size adjustments. Specifically, for a co-scheduled user employing a modulation order $Q_k$ with constellation size $M_k = 2^{Q_k}$, expanding the parent node necessitates calculating the PED for legitimate candidates strictly constrained by local geometry. This boundary-dependent evaluation and sorting process yields a degraded node-expansion computational order of $\mathscr {O}(\sqrt{M_k})$. Consequently, directly applying conventional SD to multi-user blind detection faces severe processing latency bottlenecks, especially for ultra-high-order modulations.

##### Proposition 1

**(Strictly** $\mathscr {O}(1)$ **Node-Expansion Complexity).** *Given the adaptive lattice transformation driven by the scaling matrix* $\boldsymbol{\alpha }$, *the computational complexity of the node expansion during the proposed zigzag enumeration is strictly bounded by* $\mathscr {O}(1)$, *completely independent of the heterogeneous modulation orders* $Q_k$.

##### Proof

As analyzed above, standard SD yields an expansion complexity of $\mathscr {O}(\sqrt{M_k})$ due to the necessity of boundary checking and local sorting. Conversely, in the proposed architecture, the dynamic scaling diagonal matrix $\boldsymbol{\alpha }$ actively absorbs the geometric asymmetry prior to the QR decomposition. This mapping transforms the non-uniform complex constraints into a unified one-dimensional positive integer lattice $\mathbb {Z}^+$. Let $\hat{z}_m = L_m / R_{m,m}$ be the unconstrained continuous-domain minimum. Because the search space is normalized and the layer-specific geometric constraints are eliminated, the optimal child node minimizing the PED increment $|\Delta T_m|^2$ is mathematically equivalent to the Babai nearest plane estimate. Consequently, the optimal child is acquired instantaneously through the scalar rounding operation $z_{m}^{[1]} = \lfloor \hat{z}_m \rceil$ as defined in (31). Both the continuous bounding and the subsequent incremental shifts require no sorting or boundary verification, mathematically reducing the node-expansion complexity to exactly $\mathscr {O}(1)$ per layer. $\blacksquare$

##### Proposition 2

**(Asymptotic Optimality and Bounded Error).** *The soft-LLR output of the proposed detector is asymptotically optimal. The sub-optimality gap relative to the exact ML bound is strictly constrained by a deterministic constant, and converges to zero in the medium-to-high SNR regime.*

##### Proof

Let $L_{m,b}^{\text {exact}}$ denote the exact ML bit-LLR computed using the rigorous log-sum-exp formulation in (4), and let $L_{m,b}^{\text {prop}}$ denote the LLR computed by the proposed detector using the Max-Log approximation in (6). The sub-optimality gap is defined as $\Delta L = \left| L_{m,b}^{\text {exact}} - L_{m,b}^{\text {prop}} \right|$.

By applying the Jacobian logarithm inequality, $\max _i(x_i) \le \ln \sum _i \exp (x_i) \le \max _i(x_i) + \ln (N)$, the exact ML metric can be bounded. For the two mutually exclusive sub-lattices $\mathscr {Z}_{m,b}^1$ and $\mathscr {Z}_{m,b}^0$, the maximum approximation error induced by extracting only the minimum Euclidean distance is mathematically bounded by the cardinality of the respective search spaces:

38 $$\begin{aligned} \Delta L \le \ln \left( \left| \mathscr {Z}_{m,b}^1 \right| \right) + \ln \left( \left| \mathscr {Z}_{m,b}^0 \right| \right) \le 2 \ln \left( \frac{1}{2} \prod _{k=1}^K 2^{Q_k} \right) \end{aligned}$$

This establishes a strict, deterministic upper bound on the heuristic error. Furthermore, the metric in (6) is scaled by the noise variance. As the SNR increases ($\sigma ^2 \rightarrow 0$), the Euclidean distance of the optimal lattice point, $\frac{1}{\sigma ^2}\Vert \hat{y} - Rz \Vert ^2$, grows exponentially dominant over the sub-optimal points in the summation tail. Consequently, the finite bound mathematically converges to zero:

39 $$\begin{aligned} \lim _{\sigma ^2 \rightarrow 0} \Delta L = 0 \end{aligned}$$

Additionally, because the PED increment $|\Delta T_m|^2$ defined in (29) is strictly non-negative, the accumulated distance is a monotonically non-decreasing function. Thus, the proposed $\mathscr {O}(1)$ enumeration architecture is strictly guaranteed to converge to the exact ML performance bound within a finite number of iterations, without suffering from heuristic stagnation. $\blacksquare$

## Results

We construct a rigorous link-level simulation framework adhering to the specifications of the 3GPP Release 18 Physical Downlink Shared Channel (PDSCH). Rather than assuming idealized perfect CSI, this framework implements practical DMRS-based channel estimation. Consequently, the evaluated channel matrices inherently encapsulate practical estimation errors and fading impairments.

### Modulation classification probability of co-scheduled users

In our simulation, the received signal of the target user is interfered with by a co-scheduled user, both are configured with single-layer transmission. As illustrated in Fig. 3, the classification probability $P_c$ exhibits a monotonic rise alongside increasing SNR. Statistically, this classification accuracy is evaluated empirically via exhaustive Monte Carlo simulations. At each SNR point, it is strictly calculated as the ratio of correctly identified modulation formats ($N_{\text {correct}}$) to the total number of independent inference trials ($N_{\text {total}}$), which can be expressed as $P_c = N_{\text {correct}} / N_{\text {total}}$. The method works by minimizing the Euclidean distance between the received samples and all the closest legitimate constellation points of all possible modulation schemes. The impact of symbols employed for classification is assessed in Fig. 3. In the frequency domain, for a given SNR, classification accuracy improves as more resource blocks (RBs) are allocated to the estimation process. Utilizing additional RBs for inference not only drives up computational overhead but also constrains the flexibility to adjust modulation orders across different frequency bands. As seen in Fig. 3a, bundling 2 RBs for estimation strikes an optimal balance, achieving high classification accuracy while maintaining low complexity. In the time domain, Fig. 3b contrasts performance when using all symbols within a time slot (excluding DMRS) versus a small subset of symbols adjacent to DMRS. Results indicate that classification accuracy fails to improve as more symbols are included in a single estimation. This stems from the fact that channel estimation is dependent on DMRS: REs far from DMRS symbols offer no gain for modulation order estimation, and their utilization is instead impaired by substantial channel estimation errors. Thus, choosing symbols adjacent to DMRS for modulation classification proves to be the optimal strategy, as it curtails computational effort while ensuring adequate accuracy.

**Table 2:** Key parameters of MU-MIMO configuration in simulation evaluation.

Parameters	Case 1	Case 2	Case 3	Case 4
Duplex Mode and SCS	FDD 15 kHz SCS	FDD 15 kHz SCS	FDD 15 kHz SCS	TDD 30 kHz SCS
Channel bandwidth	10 MHz	10 MHz	10 MHz	40 MHz
Co-scheduled UE Number	1	1	1	1
Rank for target-UE + Co-UE	1+1	1+1	2+2	1+1
MIMO	2T2R	2T2R	4T4R	2T2R
Channel model	TDLC 300-100	TDLC 300-100	TDLA 30-10	TDLC 300-100
Antenna correlation	ULA medium	ULA Low	XP medium	ULA medium
MCS for the target UE	MCS 13	MCS 13	MCS 17	MCS 17

### MU-MIMO detection performance

**Fig. 4:**
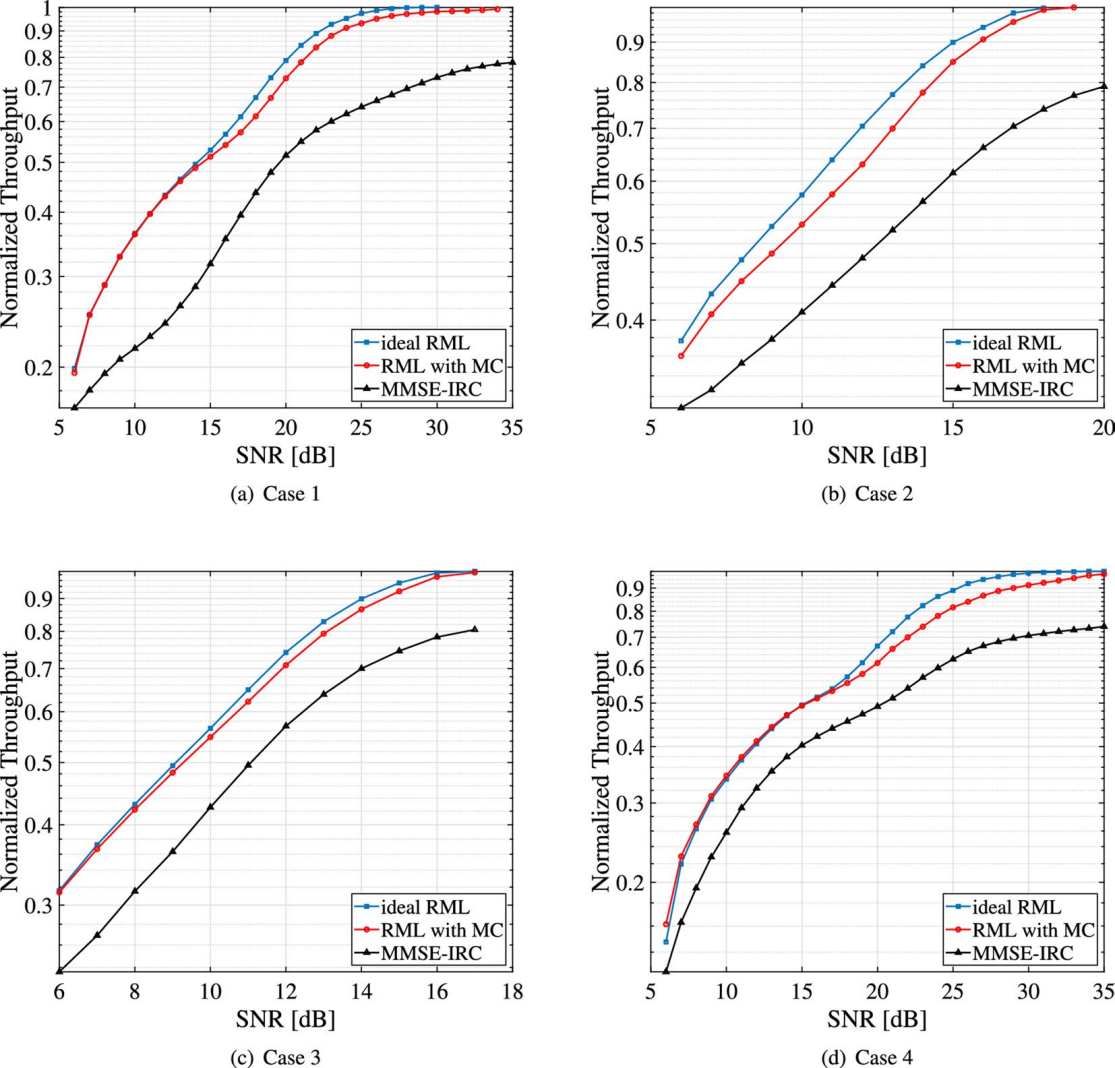
Throughput versus SNR in four types of use cases.

**Fig. 5:**
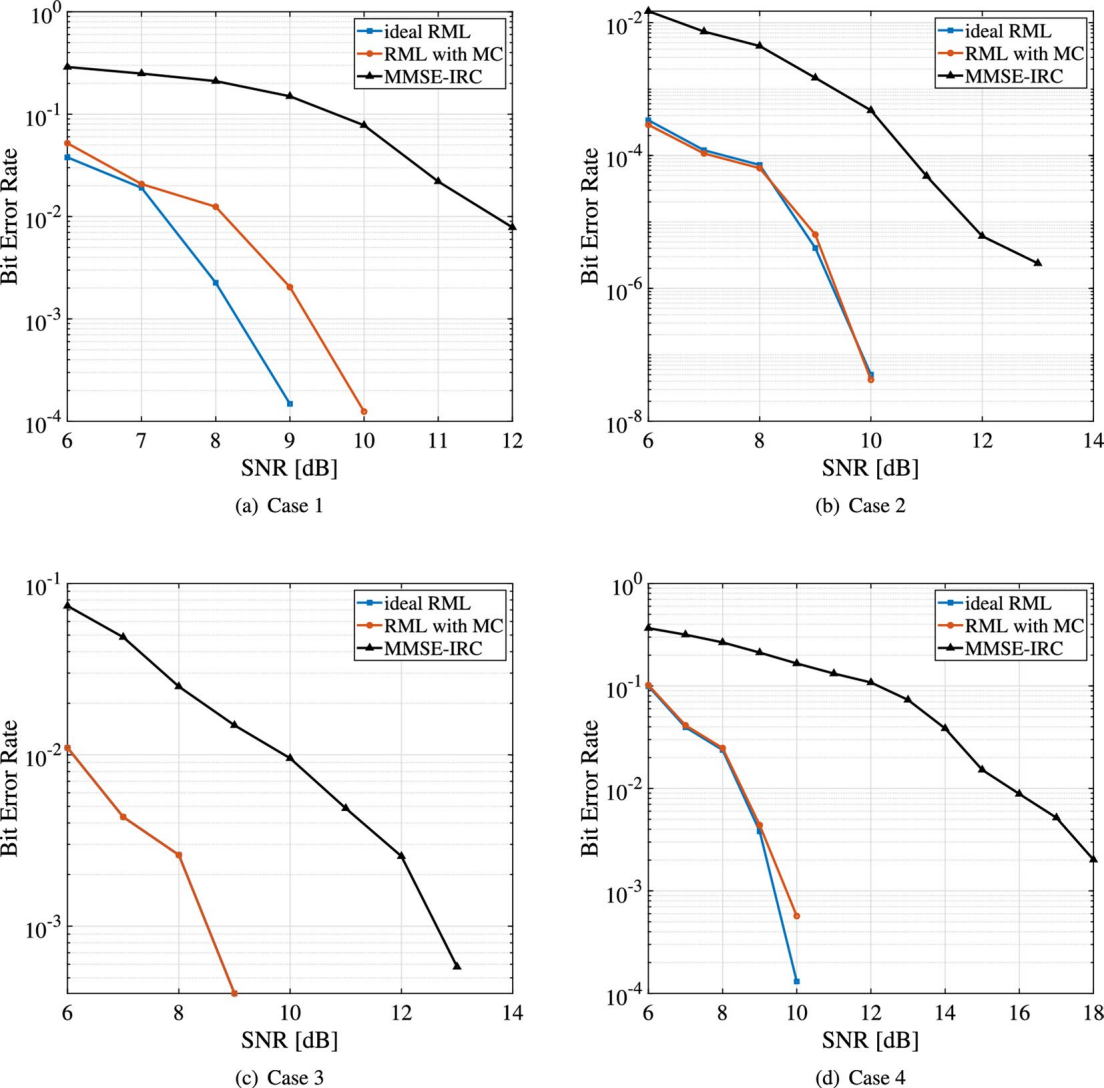
Bit Error Rate versus SNR in four types of use cases.

Subsequently, we evaluate the detection performance of the proposed algorithm across different scenarios and make comparisons with other mainstream detectors. Critical parameters, which include varying modulation orders and transmission ranks for multi-user configurations, are listed in Table 2. We evaluate performance against the 3GPP Release 15 MMSE-IRC and an Ideal RML detector. Notably, the Ideal RML curve also serves as the performance upper bound for advanced non-linear detectors, such as K-best SD, when perfectly adapted to heterogeneous modulations. Conventional advanced detectors like standard K-best SD cannot natively process blind, non-uniform MU-MIMO streams. If hypothetically supplied with perfect modulation knowledge, their throughput would asymptotically approach the Ideal RML bound presented in Fig. 4.

The results show that our proposed RML with blind MC tightly matches the Ideal RML baseline. Crucially, while a genie-aided K-best SD could theoretically match this throughput, it incurs an $\mathscr {O}(\sqrt{M_k})$ complexity due to dynamic boundary sorting. In contrast, our architecture maintains this near-optimal performance with a strictly $\mathscr {O}(1)$ complexity, as we have proved. When we focus on the $70\%$ throughput level, this level is referred to as the “operating point” and it represents the most typical load in practical communication systems. Quantitative observations can be drawn from Fig. 4 based on this. To take Case 2 as an example, the RML algorithm with MC has an operating point of 13 dB, while the MMSE-IRC detector achieves the worst performance at 17 dB. Overall, the operating point of RML with MC offers a gain ranging from 2 dB to 8 dB compared with MMSE-IRC. Viewed from the operating point, the performance degradation caused by the proposed MC algorithm is less than 1.5 dB. This arises from the proposed blind MC algorithm attaining a sufficiently high accuracy level, particularly in high-SNR conditions.

To evaluate the baseline error-correction capability of the proposed architecture, we present the un-coded Bit Error Rate (BER) performance in Fig. 5. The evaluation compares the proposed joint detector against the baseline MMSE-IRC and the Ideal RML bounds across the heterogeneous configurations outlined in Table 2. As the SNR increases, the linear MMSE-IRC detector exhibits noticeable error floors, particularly in mixed-modulation scenarios due to severe inter-user interference. Conversely, the proposed detector maintains steep waterfall curves, significantly outperforming the linear baseline and tightly bounding the Ideal RML performance.

However, a critical architectural caveat must be noted. The proposed non-linear detector is fundamentally a soft-output architecture designed to generate high-fidelity LLRs for the 3GPP LDPC channel decoder. Calculating an un-coded BER necessitates performing a hard decision on these LLRs. This hard-decision process inherently obliterates the probabilistic reliability encapsulated within the soft information, thereby underrepresenting the true capability of the detector. Consequently, while the un-coded BER effectively validates the raw symbol-level detection accuracy, the Normalized Throughput presented in Fig. 4, which natively exploits the preserved soft-LLRs during channel decoding, remains the ultimate, comprehensive metric for evaluating system-level performance.

## Conclusion

This paper explores detection mechanisms for MU-MIMO systems characterized by unknown and non-uniform modulation formats. Given that the target user lacks access to the modulation schemes of co-scheduled users, we have designed a ML-based blind modulation classification approach to address this challenge. Additionally, we put forward a MU-MIMO detection algorithm leveraging an enhanced SD framework that accommodates modulation diversity across users. Simulations conducted under representative scenarios confirm that the proposed detector outperforms multiple mainstream alternatives. While our evaluations incorporate practical channel estimation errors, extending this architecture with spatial noise whitening to mitigate severely correlated interference remains a promising direction for future work. These findings yield meaningful guidance for the evolution of advanced MU-MIMO receivers in 3GPP new release, underscoring the practical value of the work presented herein.

## Data Availability

The dataset used during the current study is available from the corresponding author on reasonable request
